# Informative STR Markers for Marfan Syndrome in Birjand, Iran

**Published:** 2012

**Authors:** Ezzat Dadkhah, Masood Ziaee, Mohammad Hossein Davari, Toba Kazemi, Mohammad Reza Abbaszadegan

**Affiliations:** 1 *Department of Human Genetics, Immunology Research Centre, Avicenna Research Institute, Mashhad University of Medical Science, Mashhad, Iran*; 2 *Birjand Hepatitis Research Centre, Birjand University of Medical Sciences, Birjand, Iran*; 3 *Ophthalmology Department, Vali-e-Asr Hospital, Birjand University of Medical Sciences, Iran*; 4 *Birjand Atherosclerosis and Coronary Artery Research Centre, Birjand University of Medical Sciences, Birjand, Iran*; 5 *Medical Genetic Research Centre (MGRC), School of Medicine, Mashhad University of Medical Sciences, Mashhad, Iran*

**Keywords:** Linkage analysis, Marfan syndrome, Microsatellite, MTS

## Abstract

**Objective(s):**

Marfan syndrome (MFS) is a severe connective tissue disorder withan autosomal dominant inheritance pattern. Early diagnosis is critical in MFS. Because of the large size of fibrillin-1 gene (*FBN1*), the uniqueness of mutations, and the absence of genotype-to-phenotype correlations linkage analysis can be very helpful for early diagnosis of MFS. In this study, eight polymorphic markers were evaluated among families related to an affected pedigree.

**Materials and Methods:**

An extended family in Birjand, Iran, with numerous cases of Marfan Syndrome in three consecutive generations, is being reported. From all consented members of these families, peripheral blood samples were collected in tubes containing EDTA. DNA extraction was performed by the conventional salting-out method. Eight STR markers were selected for linkage analysis, including four intragenic markers (MTS1, MTS2, MTS3, and MTS4) and another four flanking *FBN1* markers (D15S119, D15S126, D15S1028, and D15S143). PCR-amplified fragments were evaluated on 15% polyacrylamide gel.

**Results:**

MTS1, MTS2, and MTS3 were informative in the extended pedigree. D5S1028 was the only non-MTS marker which showed an informative diagnostic capability.

**Conclusion:**

MTS markers were informative and useful in the molecular diagnosis of Marfan Syndrome in an extended pedigree. MTS1, MTS2, and MTS3 can be used as a prenatal or presymptomatic diagnosis for all members of the extended pedigree.

## Introduction

Marfan syndrome (MFS; MIM 154700) is an autosomal dominant disorder which affects connective tissues. MFS occurs in approximately 1 in 5000 to 1 in 10000 people (1). MFS can be diagnosed by certain physical examinations. Involvement of different organ systems, such as skeletal, cardiovascular, and ocular, and the degree of severity can be variable in MFS cases. The characteristics of the severe form of Marfan Syndrome are triad symptoms, consisting of skeletal changes (long thin extremities, loose joints, and arachnodactyly), ocular changes (such as dislocation of the lens), and cardiovascular problems (such as mitral valve prolapse and regurgitation, left ventricular dilatation, cardiac failure, and pulmonary artery dilatation). MFS predisposes patients to aortic complications. Aortic root dilatation is the main cause of morbidity and mortality in patients with MFS (2, 3). The syndrome has a complete penetrance, but it shows variability in the timing of onset, tissue distribution, and the severity of clinical manifestations, both between and within affected families. The molecular analysis of the phenotypic variation began in 1991, when the fibrillin-1 gene (*FBN1*) was introduced as the site of primary mutations (4). There are three human fibrillins: fibrillin-1, fibrillin-2, and fibrillin-3, which are encoded by different genes (5). It has been shown that mutations in the* FBN1* gene (15q21.1), encoding fibrillin-1, can cause MFS (1). Fibrillin is a large glycoprotein which maintains the extracellular matrix and provides the elasticity and strength of connective tissues, including blood vessels, bones, and eyes. Mutations of the *FBN1* gene are associated with 90% of Marfan cases (6). Of the remaining 10% of these cases, some have mutations in fibrillin-2 (5q23-31). There is some evidence showing that transforming growth factor β (TGF-β), TGF-ß type I receptor (TGFBR1) and TGF-ß type II receptor (TGFBR2) genes are also related to MFS (7). *FBN1* is a 230 kb length gene, which encodes the Fibrilin-1 protein. This gene has 65 exons. Usually, major rearrangements are uncommon in the FBN-1 gene. Twelve percent of mutations are recurrent. Almost all of these mutations occur in CpG islands (8). In the Human Gene Mutation Database (HGMD), 782 different mutations have been documented for the *FBN1* gene, of which 669 are related to Marfan Syndrome. *FBN1* mutations include point mutations, gross and small deletions, gross and small insertions, and splicing mutations. According to HGMD, 66% of FBN1 mutations are missense/nonsense mutations. Approximately, 25-30% are new mutations. Missense, frameshift and splice site mutations are frequent mutations observed in MFS (9). Mutations in the* FBN1* gene could be divided into two categories: mutations causing a truncated fibrilin-1 molecule (38.6%) and the missense mutations (60.3%) (8). Except for some particular cases, no clear genotype- phenotype correlation has been detected in the *FBN1* gene (10). Since the FBN-1 gene is a large gene with 65 exons, screening and mutation detection for Marfan syndrome are not cost-effective methods. The FBN-1 mutation is not specific to Marfan syndrome, and the absence of mutation in this gene does not rule out the diagnosis of Marfan syndrome (8). Therefore, for prenatal diagnosis or presymptomatic detection of the disease in families with Marfan Syndrome linkage analysis can be useful for detecting the affected allele. In this study, eight polymorphic markers of the* FBN1* gene were used to evaluate the informativity of these markers for an extended pedigree of MFS. The informative marker(s) could be used for prenatal or presymptomatic diagnosis of the family members.

## Materials and Methods

***Patients***

The proband, the twenty-eight-year-old male in the pedigree, was referred to an ophthalmologist because of a vision problem. 

**Figure 1:**
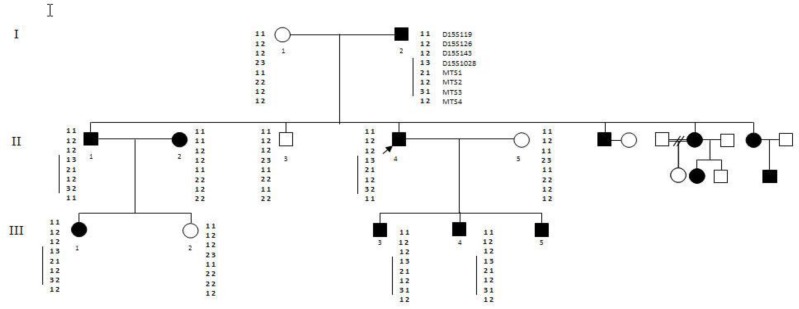
Pedigree and marker haplotypes in a family segregating MFS

The medical examination revealed a lens displacement, iridodenosis and high intraocular pressure. The patient was of tall stature and exhibited arachnodactily. He was referred to a cardiologist for further observation. The echocardiography supported the diagnosis of MFS by demonstrating aortic root dilation and mitral valve prolapse. Other members of the family were also examined for ophthalmologic and cardiologic symptoms. Diagnosis parameters were consistent with Ghent nosology (1996) (11). An extended pedigree of these relatives was recruited in this study (Figure 1). Peripheral blood samples of available members of the pedigree were collected in EDTA containing tubes, after consent forms were completed.

***Linkage analysis***

DNA extraction was performed using the conventional salting-out method. The PCR reaction mixture consisted of 1X GENETBIO PCR buffer (GENETBIO, Korea), 500 nmol/l of each PCR primer, 1.5 mmol/l MgCl_2_, 200 mol/l dNTPs and 1U of Taq DNA Polymerase (GENETBIO, Korea). A total of 50 ng DNA was used in a reaction volume of 20 µl. PCR conditions for all markers were as follows: 5 min at 95 °C followed by 35 cycles of 20 sec at 95 °C, 20 sec at an annealing temperature and 20 sec at 72 °C followed by 5 min at 72 °C as final extension, with maximum heating and cooling settings in the Techne Thermal Cycler (Techgene, Techne, UK). Eight primer sets were selected for linkage analysis, D15S119, D15S126, D15S1028, D15S143, MTS-1, MTS-2, MTS-3 and MTS-4 (Table 1) (12). Single tandem repeat (STR) segments were amplified. PCR products were electrophoresed on 2% agarose gel and stained with ethidium bromide to analyze the strength of the amplified band and the possible existence of any nonspecific bands. The PCR products were electrophoresed on 15% acrylamide gel. The allelic pattern was analyzed on the acrylamide gel and informative alleles were reported. When the affected allele could be followed in a family, it was considered an informative marker.

## Results

Patients exhibited complex symptoms, such as cardiovascular disorders and ocular and skeletal problems, that confirmed MFS. All patients were examined by an ophthalmologist and a cardiologist. The most common cardiac involvement in these patients was mitral valve prolapse and mitral regurgitation followed by the dilatation of the aortic root and aortic insufficiency. It was clarified that two sisters, two brothers, and the father of the proband's family were also afflicted with MFS. The mother and one of the brothers were healthy. There were thirteen affected MFS individuals in this pedigree of three generations (Figure 1). MTS-1, MTS-2, and MTS-4 are (CA)_n_ repeat markers, and MTS-3 contains the (TAAAA)n repeat (13). The informativity status of the markers is shown in Table 1. Informative markers could be found in all accessible families of this pedigree. D15S1028, MTS1, MTS2, and MTS3 were informative in this pedigree (Table 2). The mutant allele was detected using these intragenic and extragenic short tandem repeat markers.

**Table 1:** Primer sequences of eight STR markers of the *FBN1* gene

	Primer	Sequence	Size (bp)	Annealing temperature
1	MTS-1F	5'-CAACAAAGAAGGAGAAACAG-3'	128-146	57
2	MTS-1R	5'-GACAATGTATTCCAGAGGC-3'		
3	MTS-2F	5'-GTAGTTGTTATCTTGCAGA-3'	137-165	59
4	MTS-2R	5'-CTGCCCTCTAGGACTCTAAG-3'		
5	MTS-3F	5'-GAGTACATAGAGTGTTTTAGGG-3'	182-197	57
6	MTS-3R	5'-CCTGGCTACCATTCAACTCCC-3'		
7	MTS-4F	5'-GATGTCCCTATTGCCATCACCAC-3'	112-128	61
8	MTS-4R	5'-CCTGTGCAGGGTAAGACAAG-3'		
9	D15S119F	5'-ACTTTTGTGCCATTTAGAGATT-3'	185-197	56
10	D15S119R	5'-AACAGAAAATCCGTAACATAACATA-3'		
11	D15S126F	5'-GTGAGCCAAGATGGCACTAC-3'	188-218	56
12	D15S126R	5'-GCCAGCAATAATGGGAAGTT-3'		
13	D15S1028F	5'-GAACTGTGCTCTGTGCTC-3'	171-187	50
14	D15S1028R	5'-TGTCCTGAAATTCCCAAC-3'		
15	D15S143F	5'-CTAAGGAGGCAACAGCAAAG-3'	189-199	59
16	D15S143R	5'-ATGTAAAGACTGGTATCTGTAGCAC-3'		

**Table 2:** The informativity of eight STR markers in the pedigree with Marfan syndrome

STR	Informativity
D15S119	Noninformative
D15S126	Noninformative
D15S143	Noninformative
D15S1028	Informative
MTS1	Informative
MTS2	Informative
MTS3	Informative
MTS4	Noninformative

## Discussion

Marfan Syndrome is a clinically diagnosed disease. It has no treatment and, without any intervention, the patients may survive approximately 37 years (14). However, if intervention and attentive follow-up are provided, a normal life span is possible (6). The prognosis of MFS patients is determined by the extent and severity of the cardiovascular disorders (15). Besides the physical frustrations, patients with MFS have many psychological challenges. Living with a genetic disorder like Marfan Syndrome can exert social and emotional stress along with financial costs. Studies showed that the quality of life of the MFS patients was represented as poor. It was also clarified that 20% of patients with MFS suffer from depression (16). Mutational analysis using techniques such as single-strand conformation polymorphism (SSCP), denaturating high performance liquid chromatography, and direct sequencing can be employed for the genetic diagnosis of MFS. However, these techniques need extra money and time. In addition, only 8-30% of mutations can be diagnosed (17, 18). Most families with MFS have private mutations (19).

Using intragenic markers for linkage analysis can be a supplementary method for the MFS diagnosis of families, who have young members or show diverse or incomplete clinical symptoms (13). Moreover, molecular methods can be beneficial for prenatal diagnosis or presymptomatic individuals (12). A study in 1994 introduced four intragenic microsatellite markers for the linkage analysis of MFS: MTS1, MTS2, MTS3, and MTS-4 (20). They are located in introns 1, 5, 28, and 43, respectively. As these markers do not cover the entire gene, another group of researchers developed four other microsatellite markers spanning from the 5’ end of the gene to the intron 1 and from the intron 43 to the 3’ end of the gene. A restriction dimorphism in 3’-UTR was also employed. Their research showed that the combination of these eight microsatellite markers and the restriction dimorphism of 3’-UTR could cover complete haplotype heterozygosity in a population of 50 unrelated individuals. This panel offered a complete informativity for prenatal and presymptomatic diagnosis (12). The arrangement of these markers is shown in Figure 3. Lee *et al*, 2005 used four intragenic markers, MTS-1, MTS-2, MTS-3, and MTS-4, for linkage analysis in six families with 18 MFS patients. It was shown that MTS-2 was fully informative in four families, MTS-4 in two families and MTS-3 in just one family. In the Lee *et al* study, the MTS-1 was not informative in any family (13). Mottes M *et al* (2000) evaluated five polymorphic markers, MTS-1 to MTS-4 and 3’-UTR RsaI polymorphic site, on 50 unrelated Italian subjects. It was indicated that the MTS-1 marker did not have any significant linkage disequilibrium with the three others, MTS2, MTS3, and MTS4, even though they appeared closely linked to each other. Mottes M *et al* reported that these polymorphic markers were very effective for the identification of the disease haplotype. The informative markers were found in all 12 families, except for one (21).

**Figure 2:**
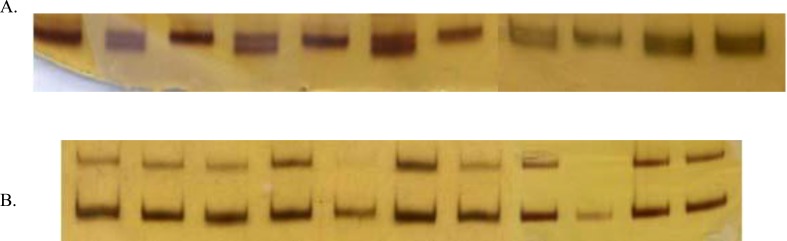
MTS1 as an informative marker (A) and D15S143 as a non-informative marker (B). From left to right: I1, I2, II3, II1, II2, III1, III2, II4, II5, III3, III4.

**Figure 3:**
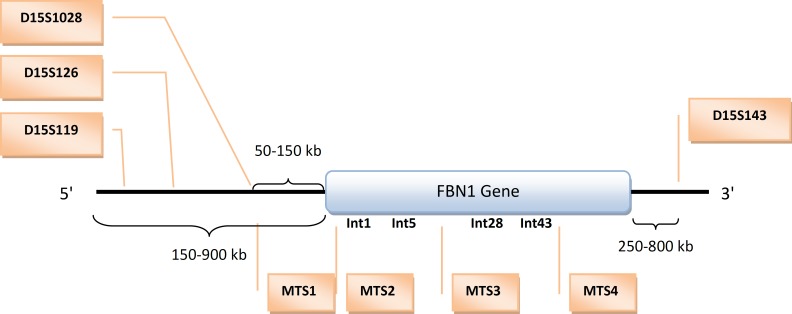
Position of polymorphic markers within and flanking the *FBN1* gene    (12)

## Conclusions

In the current study of a Birjand extended pedigree, MTS markers were more informative, compared to other markers. MTS-1, MTS-2, and MTS-3 were the common informative markers for the families, and they could be used for the prenatal or presymptomatic diagnosis in this pedigree. Selected extragenic markers did not exhibit any great informative advantage for the pedigree in this study.
